# An omics-based strategy using coenzyme Q10 in patients with Parkinson’s disease: concept evaluation in a double-blind randomized placebo-controlled parallel group trial

**DOI:** 10.1186/s42466-019-0033-1

**Published:** 2019-08-23

**Authors:** Jannik Prasuhn, Norbert Brüggemann, Nicole Hessler, Daniela Berg, Thomas Gasser, Kathrin Brockmann, Denise Olbrich, Andreas Ziegler, Inke R. König, Christine Klein, Meike Kasten

**Affiliations:** 1 Institute of Neurogenetics, University of Luebeck, Maria-Goeppert-Str. 1, 23562 Luebeck, Germany; 2 Department of Neurology, University Medical Center Schleswig-Holstein, Luebeck, Germany; 3 Department of Psychiatry, University Medical Center Schleswig-Holstein, Luebeck, Germany; 4 Center for Clinical Trials, University of Luebeck, Luebeck, Germany; 5 Institute of Medical Biometry and Statistics, University of Luebeck, Luebeck, Germany; 6 Department of Neurology, Christians-Albrechts-University, Kiel, Germany; 7 Department of Neurodegeneration, Hertie-Institute of Clinical Brain Research, Tübingen, Germany; 8 German Center for Neurodegenerative Diseases (DZNE), Tübingen, Germany; 9 School of Mathematics, Statistics and Computer Science, University of KwaZulu Natal, Pietermaritzburg, South Africa; 10 StatSol, Lübeck, Germany

**Keywords:** Parkinson’s disease, Coenzyme Q10, Personalized medicine, *PINK1*, *Parkin*, Mitochondrial dysfunction

## Abstract

**Background:**

This study focuses on genetically stratified subgroups of Parkinson’s disease patients (PD) with an enrichment of risk variants in mitochondrial genes,who might benefit from treatment with the “mitochondrial enhancer” coenzyme Q10 (156 mg coenzyme Q10/d [QuinoMit Q10® Fluid] over six months). The study will be performed in a double-blind, randomized, and placebo-controlled parallel group manner.

**Methods:**

PD patients will be specifically identified and assigned to treatment groups stratified by their genetic “mitochondrial risk burden” and consequently expected mitochondrial dysfunction and treatment response to coenzyme Q10 (homozygous or compound heterozygous *Parkin/PINK1* mutation carriers [P++], heterozygous *Parkin/PINK1* mutation carriers [P+], “omics” positive [omics+], and “omics” negative PD patients [omics-]). The primary endpoint is the change in motor symptoms over six months (as measured by the change in the motor subscore of the MDS-UPDRS). Secondary clinical endpoints include motor fluctuations, non-motor symptoms, results of magnetic resonance imaging of brain energy metabolism (31P-magnetic resonance spectroscopy imaging), and changes in structural and functional brain anatomy (MRI).

**Perspective:**

This study may be a first step towards a successful prediction of treatment response based on the genetic status of PD patients and translate progress in molecular genetics into personalized patient care. Further, magnetic resonance spectroscopy imaging may help quantify increased energy supply objectively and within a brief time after the start of treatment. Therefore, the potential of MRSI also for other studies addressing brain energy metabolism may will be assessed.

**Trial registration:**

This study was registered at the German Clinical Trial Registry (DRKS, DRKS00015880) on November 15th, 2018.

## Background

Despite rapid advances in Parkinson’s disease (PD) research, in particular in the elucidation of etiologic contributions, no disease-modifying therapy has become available to date, and the translation of these advances into improvements in patient care has proven challenging [9]. Although the treatment of motor symptoms is highly effective and can lead to a significant improvement over many years, it remains purely symptomatic and becomes increasingly difficult in the later course of the disease. Since the discovery of the first monogenic forms, it is commonly known that PD is etiologically heterogeneous [1], and there is no doubt that both genetic and environmental factors contribute to the multifactorial genesis. Although the first personalized medicine approaches through gene-specific therapeutic strategies are emerging, there remains a pressing need for disease-modifying approaches. The importance of a disease-modifying therapy is undisputed, and the heterogeneity of the disease on both a clinical and an etiological level has been demonstrated. Previous approaches employing neuroprotective therapies have yielded disappointing and heterogeneous results. Two main hypotheses are posed to explain these findings:

1. Due to a long prodromal phase, the underlying pathophysiological process is already advanced once first motor symptoms occur, therefore and, thus, any treatment in the symptomatic phase of PD may exert only limited influence on disease progression.
2. Given the heterogeneity of the disease, specific pathophysiology-based treatment approaches for subgroups are required.

The clinical study presented here focuses on the second point and aims to evaluate the efficacy of coenzyme Q10 in a specific subgroup of PD patients. Several clinical trials with coenzyme Q10 in PD patients have already been performed. Published results on the efficacy of coenzyme Q10 given to unselected PD patients are heterogeneous arguing that a global efficacy for all PD patients seems questionable [2, 8]. Coenzyme Q10 has antioxidant properties, which could mediate global efficacy, and additionally acts as a mitochondrial electron transporter, which can mediate improvement in mitochondrial function [10]. Disturbances of mitochondrial function have been proven for hereditary PD caused by mutations in the genes *Parkin* and *PINK1* [5]. The central hypothesis of this study is that coenzyme Q10 is particularly effective for PD patients with expected mitochondrial dysfunction. For this purpose, PD patients will be stratified based on their genetically determined expected mitochondrial dysfunction.

## Methods

### Aim of the trial

The current understanding of the genetic etiology of PD will be used to identify individual patients who would most likely benefit from the aforementioned specific therapy. Coenzyme Q10 as a “mitochondrial enhancer” will be tested in a randomized, double-blind, placebo-controlled parallel group trial. PD patients will be assigned to genetically defined groups stratified by their potential “mitochondrial burden” assuming that individuals with a “higher mitochondrial burden” will likely respond to coenzyme Q10 (homozygous or compound heterozygous *Parkin/PINK1* mutation carriers [P++] > heterozygous *Parkin/PINK1* mutation carriers [P+] > “omics” positive [omics+] and > “omics” negative PD patients [omics-]). The classification of the potential study participants into one of the two omics groups is based on the individual mitochondrial genetic profile. This profile describes PD patients with the highest (omics+) or lowest (omics-) cumulative burden of common genetic variants in genes related to mitochondrial function. This cumulative burden is expressed by a risk score based on eight established single nucleotide polymorphisms (SNPs) for PD (Table 2) [6]. These SNPs have been selected because they are increasing the risk for PD and are functionally linked to mitochondrial homeostasis. This score has been developed within a BMBF-funded collaborative project (MitoPD, 031A430B).

### Study description and study design

**Fig. 1:**
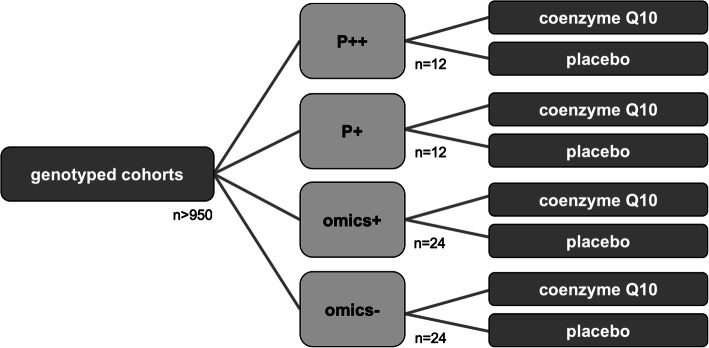
Treatment groups and their respective size

**Fig. 2:**
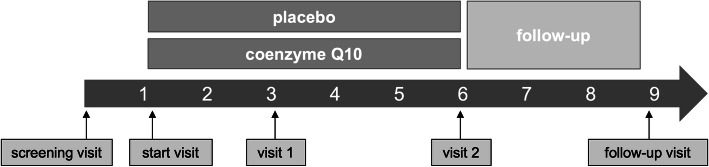
Overview on the time schedule of this clinical trial. The intermittent phone calls are not shown for better readability

The presented study is a multicenter, double-blind, controlled and randomized Phase II study in parallel group design, investigating the treatment of PD with coenzyme Q10 (156 mg/d QuinoMit® Q10 Fluid from MSE Pharmazeutika) in addition to best medical treatment compared to placebo. In each participating study center, potential study participants are examined for their genetic profile before inclusion in the study and assigned to one of the four genetic groups depending on their profile (see Fig. 1). Within the genetic groups, randomization to the experimental group or placebo control group is performed at a ratio of 1:1. The study duration per patient will be six months plus a three-month follow-up period (see Fig. 2). The primary endpoint is evaluated according to the Intention-to-treat (ITT) principle. This is practically implemented with the Full Analysis (FA) dataset (ICH E9, 1998), i.e., all registered patients who are included in the study and randomized are included in the analysis in the treatment group to which they were randomized. For the evaluation of safety, all registered and randomized patients who have received the investigational product (QuinoMit® Fluid or placebo) at least once are considered. The Per Protocol (PP) population is investigated in secondary analyses (sensitivity analyses). In the placebo group, this population consists of patients who have taken at least 80% of the investigational product. In the study group, the PP population consists of patients who took at least 80% of the investigational product and additionally had a coenzyme Q10 plasma level of at least 2.5 μg/ml at follow-up times three months and six months after randomization. The primary hypothesis of the study is that the motor symptoms of patients receiving coenzyme Q10 (QuinoMit Q10® Fluid) differ from those receiving placebo in the form of a linear difference in effect between omics-, omics+, P+, and P++.

### Arms and interventions

It is being investigated whether an improvement in motor function and mitochondrial bioenergetics can be achieved by administering 156 mg coenzyme Q10/d (QuinoMit Q10® Fluid) over six months compared to placebo. Coenzyme Q10 is approved as a dietary supplement but at a lower dosage. The substance has already been tested in clinical trials in PD patients but with less specified hypotheses.

### Outcome measures

**Fig. 3:**
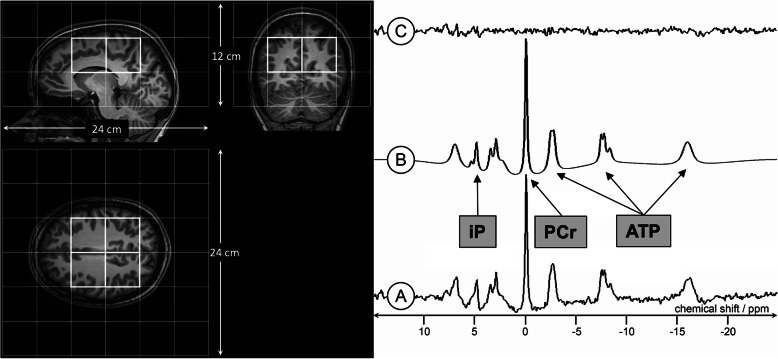
31P-Magnetic resonance spectroscopy imaging for the assessment of in vivo mitochondrial bioenergetics. 31P-MRSI spectra of a representative subset of brain parenchyma will be taken using a double-tuned P-headcoil (Advanced Imaging Research, Cleveland, Ohio). To attain sufficient relaxation of the phosphorus metabolites, a repetition time of 4500 ms will be used together with a three-dimensional chemical shift imaging sequence (6 × 5 × 3 voxel, 6 kHz bandwidth, 1024 data points, 8:51 min measuring time). The analysis procedures will follow an updated version of an already published protocol with an optimization of data acquisition. Peak positions and intensities will be calculated with the AMARES algorithm. We will examine adenosine triphosphate (ATP) and phosphocreatine (PCr), which reflects the overall high-energy phosphate turnover. PCr represents a high-energy reservoir linked to ATP in a bidirectional reaction in which ATP is formed by PCr and vice versa. In addition to PCr and ATP, the ratios of PCr/inorganic phosphate (iP) and ATP/Pi will be evaluated as an indicator of intracellular energy status within the scope of this study. A: 31P-MRSI spectrum. B: model fit on 31P-MRSI spectrum. C: background noise

The primary endpoint of the study is the change severity of PD motor symptoms after six months of treatment. The primary endpoint is measured as the difference between Part III of the revised version of the Unified Parkinson’s Disease Rating Scale (MDS-UPDRS) six months after randomization and baseline measurement. The minimally clinically relevant difference of the UPDRS III score in PD patients has been investigated in many studies providing heterogeneous results. The type I-error is set to 5% (two-sided). No previous study has taken the genetic status of PD patients into account when investigating the efficacy of coenzyme Q10. The parameters used for the power analysis are based on the work of Shulman et al. [7]. If the clinically relevant difference in the UPDRS Part III in the P++ group is large (mean = 38, standard deviation (SD) = 13.4) and there is no difference in the omics- group (mean = 27.2, SD = 13.4), while the P+ group and the omics+ group have intermediate values (mean = 34.4 and 30.8 with both SD = 13.4, respectively, the power to detect a negative slope of the interaction in the linear regression is 56% if 12, 12, 24, and 24 patients are randomized 1:1 within the four genetic groups P++, P+, omics+, and omics-. If the effect size is 1, which corresponds to a linear trend and a mean UPDRS III of 40.6 in the P++ group, the power of the study increases to 76%. We do not expect any study-related loss of patients (lost to follow-up) for the monogenetic groups (P++ and P+), as these patients have been in regular contact with the trial centers for several years. For the two omics groups, we expect patients lost to follow-up of 5%. This was the median in the four randomized clinical trials of coenzyme Q10 in PD patients conducted to date [4]. The work of Goetz et al. [3] compares the MDS-UPDRS with the original UPDRS and reports a high correlation between scales, especially for the motor part III (r = 0.96, R2 = 0.92). The case number determined for the UPDRS Part III is therefore adjusted by the factor 1/R2, resulting in a case number of approximately eighty-four patients, with approximately fourteen patients each in the two monogenic groups and approximately twenty-eight patients each in the two omics groups. Secondary clinical endpoints include quality of life (Parkinson’s Disease Quality of Life Questionnaire [PDQ39]), depression (Beck’s Depression Inventory II, BDI II), motor fluctuations and disease-related complications (MDS-UPDRS IV), daily activities (MDS-UPDRS II), fatigue (Fatigue Severity Scale [FSS]), sleepiness (Epworth’s Sleepiness Scale [ESS] and Parkinson’s Disease Sleep Scale 2 [PDSS2]), and cognitive function (Montreal Cognitive Assessment [MoCA]) (see Table 1). Secondary endpoints also include magnetic resonance imaging of brain energy metabolism (31P-magnetic resonance spectroscopy imaging [MRSI], see Fig. 3), as well as changes in structural, and functional brain anatomy (MRI). The secondary efficacy endpoints are evaluated in an explorative manner analogous to the primary endpoint. All relevant safety endpoints are described descriptively with suitable statistical measures.

**Table 1:** Overview on clinical assessments for each study visit

time (months)	visit	clinical assessments
−3-0	screening	MDS-UPDRS III Timed up and go test 10-m-walk test vital signs (safety) blood analyses MDS-UPDRS I, II, and IV PDQ39, BDI II, FSS, ESS, PDSS2, MoCA informed consent check of inclusion/exclusion criteria
0	start visit	MDS-UPDRS III Timed up and go test 10-m-walk test vital signs (safety) blood analyses MDS-UPDRS I, II, and IV PDQ39, BDI II, FSS, ESS, PDSS2, MoCA MRI/MRSI
1	phone call 1	interview on AEs/SAEs, inquiry on self-ratings (diary cards)
2	phone call 2	interview on AEs/SAEs, inquiry on self-ratings (diary cards)
3	visit 1	MDS-UPDRS III Timed up and go test 10-m-walk test vital signs (safety) blood analyses MDS-UPDRS I, II, and IV PDQ39, BDI II, FSS, ESS, PDSS2, MoCA
4	phone call 3	interview on AEs/SAEs, inquiry on self-ratings (diary cards)
5	phone call 4	interview on AEs/SAEs, inquiry on self-ratings (diary cards)
6	visit 2 (end of study treatment)	MDS-UPDRS III Timed up and go test 10-m-walk test vital signs (safety) blood analyses MDS-UPDRS I, II, and IV PDQ39, BDI II, FSS, ESS, PDSS2, MoCA MRI/MRSI
9	follow-up visit	MDS-UPDRS III Timed up and go test 10-m-walk test vital signs (safety) blood analyses MDS-UPDRS I, II, and IV PDQ39, BDI II, FSS, ESS, PDSS2, MoCA MRI/MRSI

*AE* adverse event, *BDI II* Becks Depression Inventory II, *ESS* Epworth’s Sleepiness Scale, *FSS* Fatigue Severity Scale, *MDS-UPDRS* Movement Disorders Society Unified Parkinson’s Disease Rating Scale, *MoCA* Montreal Cognitive Assessment, *MRI* Magnetic Resonance Imaging, *MRSI* Magnetic Resonance Spectroscopy Imaging, *PDQ39* Parkinson’s Disease Quality of Life Questionnaire (39 items), *PDSS2* Parkinson’s Disease Sleep Scale 2, *SAE* serious adverse events

### Eligibility criteria

Inclusion criteria consist of confirmed PD diagnosis according to the UK Brain Bank criteria, successful genotyping and assignment to the treatment groups, stable antiparkinsonian medication (for at least four weeks before study enrollment), age of 18 years or above, and written informed consent. Exclusion criteria include: comorbidities affecting the capability of giving informed consent (e. g. severe dementia [MMSE of 24 points or below], psychosis, acute severe depression), atypical or secondary parkinsonism, unstable Parkinson medication, pregnancy and/or breastfeeding, desire to have children, unwillingness to use contraception (if study participant is not sterile/in menopause), supplementation of coenzyme Q10 up to three months prior to baseline visit, known intolerance to coenzyme Q10, medication with thyroid hormones, simultaneous intake of vitamin K antagonists (such as warfarin), simultaneous intake of beta blockers, epilepsy, structural brain damage, allergy to soy, simultaneous participation in another clinical trial, and known severe liver or kidney dysfunction.

### Genetic stratification

For analyses, the study population is divided into four genetic subgroups (P++, P+, omics+ and omics-). Patients in the P++ group carry biallelic mutations (homozygous or compound heterozygous) in the *Parkin* or *PINK1* gene. Patients in the P+ group carry a single, i.e. heterozygous mutation in the *Parkin* or *PINK1* gene. The classification of the patients into the two omics groups is done via a simple sum score and is calculated as follows (see Table 2):

$$ {\sum}_{i=1}^9 number\ of\ effect\ allel es\ {SNP}_i\cdot \mathit{\ln}\ \left( odds\ ratio\ effect\ allel\ {SNP}_i\right)+1,7181 $$

**Table 2:** SNPs taken a genome-wide association study on PD used for stratification of patients (omics+/− groups)

SNP	Locus	Effect allele	MAF	Odds Ratio
rs329648	MIR4697	T	0,46	1,15
rs34311866	TMEM175-GAK-DGKQ	G	0,14	1,40
rs11868035	SREBF1-RAI1	A	0,49	0,97
rs14235	BCKDK-STX1B	A	0,36	1,19
rs11060180	CCDC62	G	0,25	0,90
rs71628662	GBA-SYT11	T	0,01	0,40
rs199347	GPNMB	C	0,48	0,97
rs12637471	MCCC1	A	0,34	0,67

SNPs were taken from the study of Nalls et al. [6]. Only SNPs were taken into account with an in silico annotation to mitochondrial homeostasis. SNP: single nucleotide polymorphism. MAF: mean allele frequency

The number of effect alleles (coding 0 = no effect allele, 1 = one effect allele and 2 = two effect alleles) and the weighting of the respective SNP with the effect size of the effect allele, measured with the ln of the odds ratio, are incorporated into this score from each risk SNP. If the calculated risk score for a patient exceeds a threshold value of + 0.30, this patient is assigned to the omics+ group. If a patient has a risk score of less than − 0.30, an assignment to omics- is made. Patients with a risk score between − 0.30 and + 0.30 do not belong to one of the two “extreme” omics groups. These patients are not included in the study if they are not heterozygous, homozygous or compound heterozygous for mutations in the *Parkin* or *PINK1* gene. In patients who are assigned to one of the two omics groups, mutations in the *PINK1* or *Parkin* gene will be excluded. The thresholds as mentioned above were determined to represent the 20 and 80% quantiles in our cohorts in which the score has been established. A number of the study participants’ samples will be genotyped twice in order to ensure the reproducibility of genotyping. In this way, the agreement can be determined SNP-specifically and overall. The intra-class correlation coefficient is used as a measure of agreement. It is suspected that the four groups differ in the degree of mitochondrial dysfunction, with increasing dysfunction from omics- via omics+ and P+ to P++. Accordingly, it is assumed that the efficacy of coenzyme Q10 treatment increases from omics- to P++. The aim is therefore to investigate the efficacy of Q10 as a function of genetic background. For each genetic subgroup, all secondary endpoints are analyzed exploratively.

## Contacts

Department of Neurology, University Medical Center Schleswig-Holstein, Ratzeburger Allee 160, 23538 Lübeck, Germany.

Department of Neurology, University Medical Center Schleswig-Holstein, Arnold-Heller-Straße 3, 24105 Kiel, Germany.

Department of Neurodegeneration, Hertie-Institute of Clinical Brain Research, Tübingen, Hoppe-Seyler-Straße 3, 72076 Tübingen, Germany.

## Perspective

This study is an essential step to elucidate at least one therapeutically relevant disease mechanism for a subset of PD patients. Also, it is one of the first approaches to successfully base treatment decisions on the genetic status of PD patients and translate progress in molecular genetics into personalized patient care. If this proof-of-principle study is successful, future questions will address whether a potential benefit is sustained and whether the improvement is a mere symptomatic effect associated with improved energy metabolism or due to neuroprotective actions. It would also be of great interest to see whether the proposed omics-score helps to stratify PD patients or whether further improvements are needed to identify mitochondrial subgroups of PD patients (e.g., by selection/combination of other SNPs or functional assays) to impact more significantly on PD patient care. Due to its noninvasive nature, MRSI can be applied repeatedly, pre- and post-intervention in a proof-of-concept clinical trial. 31P-MRSI measures will most likely be the most sensitive marker to objectify alterations of mitochondrial bioenergetics in vivo. The combination of personalized treatment choices and concomitant neuroimaging markers may provide a substantial opportunity to personalize treatment choices for PD.

## Data Availability

Data sharing is not applicable to this article as no datasets were generated or analyzed for this manuscript.

## Abbreviations

AE: Adverse event
ATP: Adenosine triphosphate
BDI II: Becks Depression Inventory II
DRKS: German Registry for Clinical Trials
ESS: Epworth’s Sleepiness Scale
FA: Full analysis
FSS: Fatigue severity scale
iP: Inorganic phosphate
ITT: Intention to treat
MAF: Mean allele frequency
MDS-UPDRS: Movement Disorders Society Unified Parkinson’s Disease Rating Scale
MoCA: Montreal cognitive assessment
MRI: Magnetic resonance imaging
MRSI: Magnetic resonance spectroscopy imaging
omics-: Treatment group with the lowest polygenic score
omics+: Treatment group with the highest polygenic score
P +: Heterozygous *Parkin/PINK1* mutation carriers
P++: Homozygous/compound heterozygous *Parkin/PINK1* mutation carriers
PCr: Phosphocreatinine
PD: Parkinson’s disease
PDQ39: Parkinson’s Disease Quality of Life Questionnaire (39 Items)
PDSS2: Parkinson’s Disease Sleep Scale 2
PP: Per Protocol population
SAE: Serious adverse event
SD: Standard deviation
SNP: Single nucleotid polymorphism
